# O02 Evaluating the application of antimicrobial stewardship in patients referred to the sepsis team

**DOI:** 10.1093/jacamr/dlaf118.002

**Published:** 2025-07-14

**Authors:** Hibah Mirza, Karamjit Badyal, Kiranmai Bhatt, Aiden Plant

**Affiliations:** Walsall Manor Hospital, Walsall, UK; Walsall Manor Hospital, Walsall, UK; Walsall Manor Hospital, Walsall, UK; Walsall Manor Hospital, Walsall, UK

## Abstract

**Background:**

Sepsis is defined as life-threatening organ dysfunction caused by a dysregulated host response to an infection. Early recognition and prompt appropriate treatment is associated with better outcomes. Where there is evidence of bacterial infection, effective antibiotics must be promptly administered. With the increasing threats of antimicrobial resistance (AMR) there is a need for a greater alignment between sepsis and antimicrobial stewardship programmes. While it is essential to administer effective antibiotics, timely blood culture sampling, selection of empirical antibiotics in line with local guidelines and appropriate de-escalation can ensure antibiotics are used appropriately. The sepsis team and antimicrobial stewardship team in a 500 bed UK NHS hospital collaborated to evaluate how effectively empirical antibiotics are initiated in patients with suspected sepsis.

**Methods:**

A sample of patients that had been referred to the sepsis team between January and May 2024 were reviewed. The indication for the antibiotic, adherence to Trust antimicrobial guidelines, blood culture sampling and results were reviewed to assess if antimicrobial stewardship principles had been followed.

**Results:**

97 patients were referred to the sepsis team in the study time frame. The leading suspected sources of infection were CAP (26%), IECOPD (12%, intra-abdominal sepsis (12%), HAP (11%) and UTI (9%). Blood cultures had been taken in 53% of patients, of those 18% had a positive culture result, *Escherichia coli* was the leading isolated organism. The timing of the blood cultures was assessed, it was found that 36% (*n*=35) of blood cultures were taken between the hours of 9 am and 5 pm and 64% (*n*=62) were outside of these hours. Antibiotics were appropriate and in line with Trust guidelines for 49% (*n*=48) of patients and inappropriate for 51% (*n*=49) of patients; this percentage figure was the same both between 9 am and 5 pm and outside of 9 am–5 pm.

**Conclusions:**

Blood cultures had not been taken in 47% of patients that were referred to the sepsis team for review, 51% of these patients did not have antibiotics prescribed in line with Trust guidelines. Although blood culture sampling had been completed in more patients outside of 9 am–5 pm working hours, adherence to Trust guidelines was the same. The initiation of antibiotics in patients with suspected sepsis is currently suboptimal and is an important target area for antimicrobial stewardship teams.

